# Genome sequence of *Hemileia vastatrix* Berk. and Br. (Race I), the causal agent of coffee leaf rust, isolate from Risaralda, Colombia

**DOI:** 10.1128/MRA.00444-23

**Published:** 2023-10-04

**Authors:** Carlos A. Ángel C., Gustavo A. Marín-Ramírez, Carlos E. Maldonado

**Affiliations:** 1 Plant Pathology, Colombian National Coffee Federation (FNC)/National Coffee Research Center (CENICAFE), Chinchiná, Colombia; 2 Plant Breeding, Colombian National Coffee Federation (FNC)/National Coffee Research Center (CENICAFE), Chinchiná, Colombia; University of California, Riverside, California, USA

**Keywords:** coffee leaf rust, *Hemileia vastatrix*, *Coffea arabica*, genomes, PacBio HiFi, Dovetail Omni-C, annotation, transposable elements

## Abstract

Coffee leaf rust, caused by the fungus *Hemileia vastatrix* (Basidiomycota; Pucciniomycota)*,* is a devastating disease spread worldwide. To improve the available genomes, we use PacBio HiFi sequencing enhanced by Dovetail Omni-C chromatin conformation capture to assemble a highly contiguous 747.98 Mb genome of an isolate collected from *Coffea arabica*.

## ANNOUNCEMENT

Coffee leaf rust (CLR), caused by *Hemileia vastatrix* Berk and Br., is the most important coffee disease, causing epidemics and yielding losses up to 80% in susceptible varieties (1, 2). Here, we present a high-quality highly contiguous whole-genome assembly of *H. vastatrix* Race I, improving the previous reported assemblies (3 – 5).

**Fig 1:**
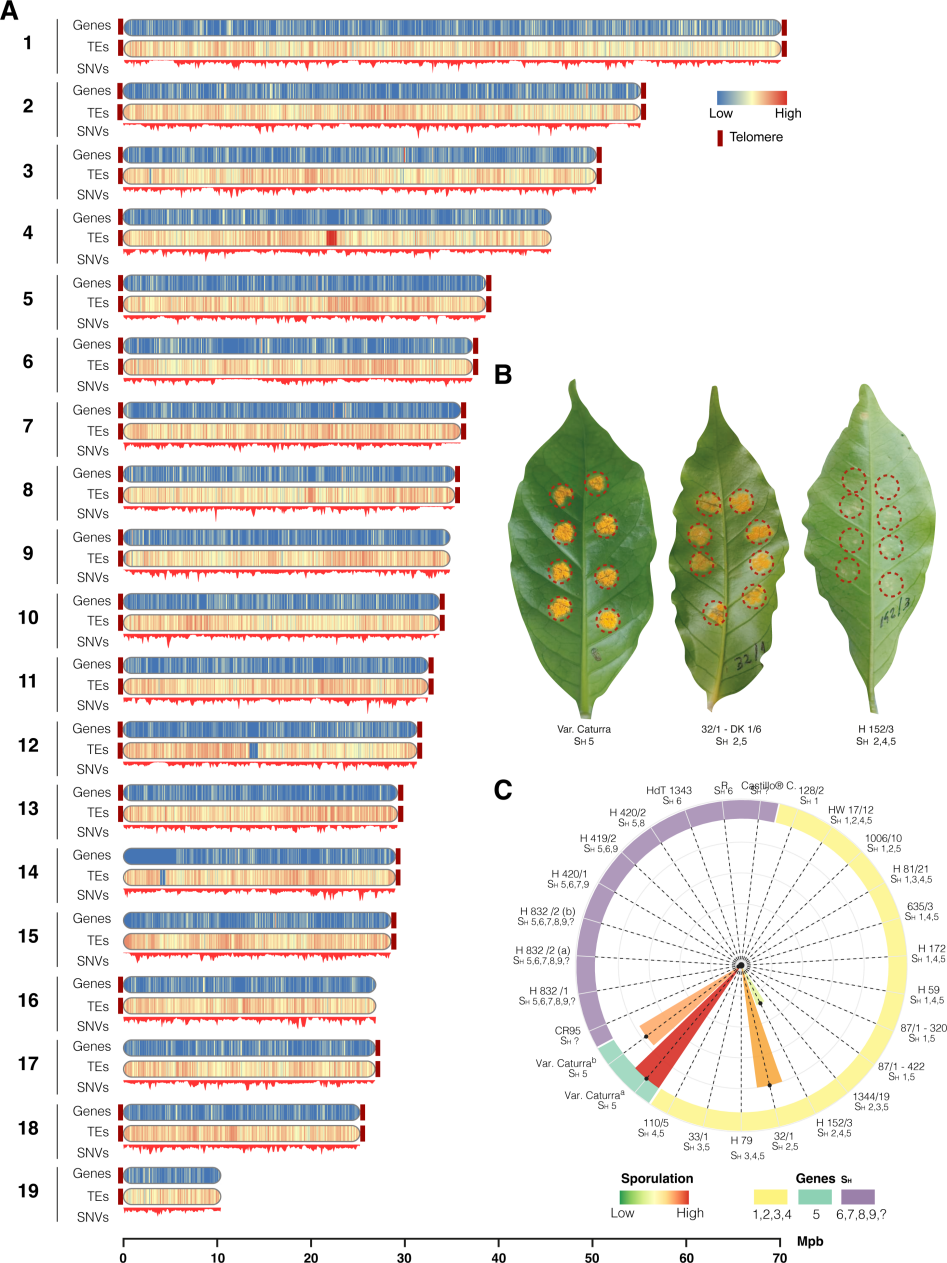
Phenotyping and genomic analysis of *Hemileia vastatrix* Race I. (**A**) Features of the 19 largest scaffolds including telomere positions, heat maps of genes and transposable elements in 10 Kbp windows, and distribution of single nucleotide variants (SNVs) from polishing. (**B**) Phenotypic characterization of the *H. vastatrix* isolate inoculated on hosts carrying different CLR resistance genes (*SH*). (**C**) Percentage of sporulated inoculation sites on 26 coffee differential clones; Var. Caturra^a^ and Var. Caturra^b^ are the inoculation positive controls incubated under controlled and uncontrolled environment, respectively.

We isolated *H. vastatrix* from a *Coffea arabica* var. Caturra plantation in the rural area of Pereira (Risaralda, Colombia), at 4°44'46.25"N, 75°36'14.59" W. Twenty successive inoculation cycles of 40–50 days on 5-month-old plants of the Caturra variety were done to increase the urediniospore mass to obtain enough spores for DNA isolation and phenotyping. The isolate was inoculated on 26 coffee genotypes carrying different combinations of CLR resistance genes (S*H*). Compatible infection reaction was observed on carriers of the genes *SH*2 and *SH*5, assigning the isolate to Race I (6) (Fig. 1).

The DNA isolation and sequencing were performed by the Arizona Genomics Institute. High molecular weight DNA was isolated from urediniospores using a modified CTAB protocol (7). A PacBio 30 Kb library was constructed from size-selected DNA (BluePippin system) using the SMRTbell express template prep kit 2.0, and two SMRTcells 8M v1 were sequenced on a Sequel II sequencer using the SeqII 1.0 chemistry. DoveTail Genomics constructed an Omni-C library using a proximity-ligation protocol on immobilized chromatin, followed by library construction using NEBNext Ultra enzymes and Illumina-compatible adapters, followed by sequencing on an Illumina HiSeqX platform. PacBio produced 3,439,300 circular consensus sequences (HiFi reads) with an N50 of 16,166 bp, while 139.2 million paired reads of 150 bp were produced from the Omni-C library, resulting in coverage greater than 70× and 58×, respectively, for an ~ 719 Mbp genome estimated by GCE v1.0.2 (8). For polishing, 50 million DNBseq 150-PE reads, covering 21× the genome, were produced by Complete Genomics from DNA isolated using the DNeasy Plant Kit (Qiagen).

A primary genome assembly was generated by Hifiasm (9) using HiFi reads. The assembly was scaffolded by HiRise pipeline (10) using the Omni-C sequences and polished by Polca (11) using DNBseq data. Contaminants were trimmed by FCS, FCS-GX (https://github.com/ncbi/fcs/), and NCBI Genome Workbench (12). The final polished assembly had a total length of 747,980,314 bp, a consensus quality of Q40.82, and a GC content of 33.78%. Assembly statistics were obtained by QUAST 5.2.0 (13) (Table 1), reporting a BUSCO genome completeness score of 97.93% using the fungi_odb9 as lineage data set. Transposable elements were *de novo* called using the EDTA pipeline (14), which masked 81.66% of the genome. Evidence-based annotation was performed by Augustus (15, 16), calling 12,870 protein genes on the masked sequence. Telomere repeat sequences screening was done by Tapestry v1.0.1 (17), the motif CCCTAA/TTAGGG. Telomeres were found at the end of the 19 longest scaffolds, 11 of those were assembled telomere to telomere (Fig. 1).

**TABLE 1:** Genomic features and statistics of *Hemileia vastatrix* Race I after assembly, scaffolding, and polishing

	Primary assembly HiFiasm v. 0.14	Dovetail HiRise assembly	Polished assembly
Total length (bp)	775,474,960	775,477,860	747,980,110
N50	19,794,549	35,487,552	35,997,362
L50	14	9	8
N90	531,823	531,823	25,694,141
L90	55	26	18
Largest scaffold	45,329,991	71,418,495	71,415,227
Number of contigs	985		
Number of scaffolds		956	794
Number of gaps	0	29	29
GC (%)	33.91	33.91	33.78

## Data Availability

The Hv_R1_Cat_CENPAT genome assembly is available at NCBI under the BioProject accession number PRJNA912191, BioSample SAMN32232808, GenBank assembly accession GCA_030280995.1 WGS PacBio and DNBseq sequences are available on NCBI’s SRA database with the IDs SRR22911636 and SRR22911637. The evidence-based structural annotation for this assembly is available on the CENICAFE: institutional repository (https://doi.org/10.38141/10799/dataset02) as well as the transposable elements library derived from *de novo* calling (https://doi.org/10.38141/10799/dataset01).
